# The Direct Interaction between E93 and Kr-h1 Mediated Their Antagonistic Effect on Ovary Development of the Brown Planthopper

**DOI:** 10.3390/ijms20102431

**Published:** 2019-05-16

**Authors:** Yiwen Mao, Yan Li, Han Gao, Xinda Lin

**Affiliations:** College of Life Sciences, China Jiliang University, Hangzhou 310018, China; Maoyiwen0625@163.com (Y.M.); 15825533072@163.com (Y.L.); 15255296862@163.com (H.G.)

**Keywords:** *E93*, *Kr-h1*, brown planthopper, protein–protein interaction

## Abstract

The juvenile hormone (JH) signalling and ecdysone signalling pathways are crucial endocrine signalling pathways that orchestrate the metamorphosis of insects. The metamorphic process, the morphological change from the immature to adult forms, is orchestrated by the dramatic reduction of JH and downstream transcription factors. The Krüppel-homologue 1 (Kr-h1), a downstream transcription factor of the JH signalling pathway, represses *E93* expression with an anti-metamorphic effect. However, the biochemical interaction between *Kr-h1* and *E93* and how the interaction regulates ovary development, a sensitive readout for endocrine regulation, remain unknown. In brown planthopper, *Nilaparvata lugens*, we found that the downregulation of *Kr-h1* partially recovered the deteriorating effect of *E93* knock-down on metamorphosis. Dual knock down of *E93* and *Kr-h1* increased ovary development and the number of eggs laid when compared to the effects of the knock down of *E93* alone, indicating that the knock down of *Kr-h1* partially recovered the deteriorating effect of the *E93* knock-down on ovary development. In summary, our results indicated that *E93* and *Kr-h1* have antagonistic effects on regulating metamorphosis and ovary development. We tested the biochemical interaction between these two proteins and found that these molecules interact directly. Kr-h1 V and E93 II undergo strong and specific interactions, indicating that the potential interacting domain may be located in these two regions. We inferred that the nuclear receptor interaction motif (NR-box) and helix-turn-helix DNA binding motifs of the pipsqueak family (RHF1) are candidate domains responsible for the protein–protein interaction between E93 and Kr-h1. Moreover, the HA-tagged E93 and FLAG-tagged Kr-h1 were co-localized in the nucleus, and the expression of *E93* was increased when *Kr-h1* was downregulated, supporting that these two proteins may interact antagonistically. JH and ecdysone signalling are critical for the control of ovary development and pest populations. Our result is important for understanding the interactions between E93 and related proteins, which makes it possible to identify potential targets and develop new pesticides for pest management.

## 1. Introduction

The juvenile hormone (JH) signalling and ecdysone signalling pathways are two crucial endocrine signalling pathways that regulate the metamorphosis of insects, which is the morphological change from immature to adult forms and is orchestrated by the dramatic reduction in the JH level and the downstream transcription factor of the JH signalling pathway [1,2,3,4,5]. The binding of JH to the Methoprene-tolerant (Met) and Taiman (Tai) receptor complex induced the expression of a C_2_H_2_ down-stream zinc-finger transcription factor Krüppel-homologue 1 (Kr-h1) [6,7,8,9]. One key role of Kr-h1 during development is to prevent the larval–pupae transition and metamorphosis [7,8], which is also a major function of JH.

The transcription factor E93 is induced by ecdysone and broadly expressed in pupae and adult insects [10,11,12,13,14]. *E93* was first identified from *Drosophila melanogaster* and found to be induced by the prepupal ecdysteroid pulse [15]. E93 is a pipsqueak family protein, and its homologues range from *Caenorhabditis elegans* (MBR-1) [16] to humans (LCoR) [17]. Most of the insect E93 proteins contain important domains: one CtBP-interacting motif (CtBP-im), one to two nuclear receptor interaction motifs (NR-box), and one to two helix-turn-helix DNA binding motifs of the pipsqueak family (RHF) [11,16,18]. The molecular function of *E93* includes determining programmed cell death in larval salivary glands during development and transducing ecdysone signalling to induce autophagy and caspase activity in the remodelling fat body of *D. melanogaster* [14,19]. Evidence from multiple species, including both hemimetabolous insects, such as *Blattella germanica* [11,13], and holometabolous insects, such as *Drosophila melanogaster* [12,14] and *Tribolium castaneum* [11], indicated that *E93* triggers adult morphogenesis and specifies adult metamorphosis [11,13,20,21]. Krüppel-homologue 1 (Kr-h1) represses *E93* expression with an anti-metamorphic effect [11,13,20]. Indeed, the downregulation of *Kr-h1* leads to a premature phenotype, and even bypasses the pupae stage in holometabolous insects [20]; both *E93* and *Kr-h1* regulate metamorphosis [18,20].

In the notorious rice insect pest of Asian countries, brown planthopper, *Nilaparvata lugens*, JH receptor Met and its downstream transcription factor Kr-h1, as well as the transcription factor Br-C, which is downstream of ecdysone signalling, have been shown to interact with each other and regulate not only metamorphosis but also ovary development [10,22]. Although genetic and developmental studies have revealed the interaction between *Kr-h1* and *E93*, the biochemical interaction between *Kr-h1* and *E93* and how this interaction regulates ovary development, a sensitive readout for endocrine regulation, remain unknown. Here, we explored the interaction of E93 and Kr-h1 through single or dual knock down of *E93*/*Kr-h1* and observed and compared the morphological changes. Moreover, we studied the protein–protein interaction and possible correlation with transcription changes to understand the interaction between E93 and Kr-h1 and the effect on ovary development.

## 2. Results

### 2.1. Downregulation of Kr-h1 Partially Recovered the Deteriorating Effect of E93 Knock-Down on Metamorphosis

**Figure 1:**
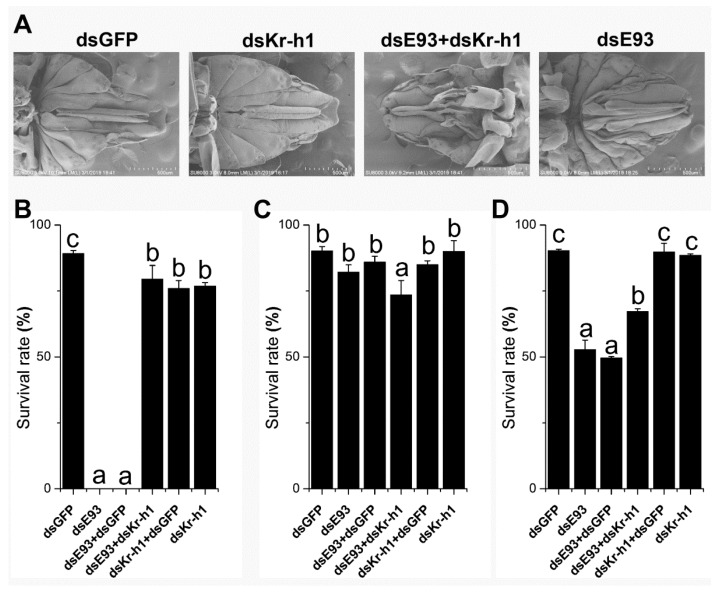
Downregulation of *Kr-h1* partially recovered the deteriorating effect of *E93* knock-down on metamorphosis. (**A**) External genitalia phenotype of brown planthoppers after RNAi of *E93* and *Kr-h1* alone or together. Transmission electron microscopy (TEM) was used to observe the phenotype of external genitalia after the eclosion of adults. Nymphs were injected at the 4th instar stage. (**B**) dsRNAs of *E93* and *Kr-h1* were injected alone or together at the 3rd instar larval stage, and the survival rate after moulting from the 3rd to 4th instar nymph stage is shown; (**C**) dsRNA of *E93* and *Kr-h1* were injected alone or together at the 4th instar nymph stage, survival rate after moulting from 4th instar nymph to 5th instar nymph is shown; (**D**) survival rate after metamorphosis from the 5th instar nymph to adult after the injection of *E93* and *Kr-h1* alone or together. Duncan’s multiple comparison was used, and different letters indicate significant differences (*p* < 0.05).

We down-regulated the expression of *E93* and *Kr-h1* by the injection of dsRNA into 4th instar nymphs. The phenotype is reminiscent of a defect in specifying adult metamorphosis (Figure 1A), especially the external female genitalia, which is either undeveloped or underdeveloped compared to that of the control injected with dsGFP (Figure 1A), and the defect is less intense in the dual knock-down (Figure 1A), suggesting the normal morphology is partially recovered. We also injected the dsRNA of *E93* into 3rd instar nymphs, which showed an 0% survival rate, indicating that the downregulation of *E93* at this stage is lethal (*p* < 0.05, Figure 1B). However, the downregulation of *Kr-h1* by the injection of dsRNA showed a survival rate of 76.9% after moulting from the 3rd to the 4th instar nymph stage (*p* < 0.05, Figure 1B), which was significantly lower than that of the control injected with dsGFP (*p* < 0.05, Figure 1B) and significantly higher than that of *E93*. Again, to our surprise, when we injected *E93* and *Kr-h1* dsRNAs simultaneously, the survival rate increased dramatically to 79.5%, although it was not significantly different from that after injecting *Kr-h1* dsRNA alone (*p* < 0.05, Figure 1B). The survival rate from the 4th instar nymph to the 5th instar nymph after dual injection was significantly lower than that after injection with dsRNA of *E93* or *Kr-h1* alone (*p* < 0.05, Figure 1C). The downregulation of *E93* at the 5th instar nymph stage showed a significantly lower survival rate (Figure 1D). The survival rate in the dual knock-down increased significantly compared to the injection of *E93* dsRNA alone but was lower than that of the *Kr-h1* knock-down (Figure 1D). Again, this finding suggested a possible antagonistic interaction between *E93* and *Kr-h1*. In summary, the survival rate after down-regulation of the expression of *E93* and *Kr-h1* in the 3rd and 5th instar nymphs indicated that *Kr-h1* partially restored the knockdown effect of *E93*.

Taken together, this experiment indicated that *E93* and *Kr-h1* may have antagonistic effects on regulating metamorphosis, especially at the 3rd and 5th instar nymph stages.

### 2.2. Downregulation of Kr-h1 Partially Recovered the Deteriorating Effect of E93 Knock-Down on Ovary Development

**Figure 2:**
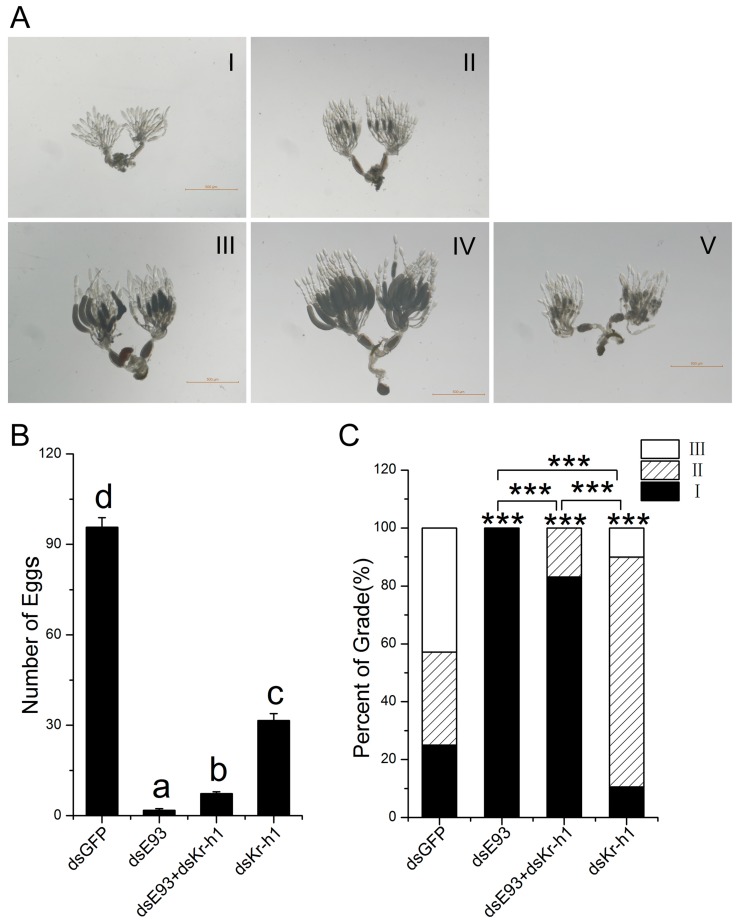
Downregulation of *Kr-h1* partially recovered the deteriorating effect of *E93* knock-down on ovary development. The 5th instar nymphs were injected with *E93* and *Kr-h1* alone or together. (**A**) ovary grades I to V,I: transparent stage, ovarian tubules are transparent, II: vitellogenesis stage, ovarian tubules are transparent to milky white, III: matured stage, milky white mature eggs appear at the base of the ovarian tubule, IV: egg-laying stage with full-developed ovary, V: Last stage of egg-laying, ovarian tubule atrophy and nearly no egg was laid; (**B**) the number of eggs per female (short-winged), Duncan’s multiple comparison was used, *p* < 0.05; (**C**) the ovary grade (short-winged). Ovary grading was carried out two days after eclosion. Ovary development of the treatment (*E93* and *Kr-h1*, alone or together) and the control dsGFP were compared, and single and dual knock down of *E93* and *Kr-h1* were also compared. dsGFP was used as a control. A chi-square test was used, ***: *p* < 0.001. The “***” immediately above the column indicates the comparison between the treatment and the control dsGFP. The comparisons among treatments were indicated as specifically by transverse bracket.

Previous studies have shown that both JH and ecdysone signalling are required for ovary development, which is actually sensitive to the disruption of endocrine signalling. In addition, we have shown that the downregulation of *Kr-h1* delayed ovary development [6]. The number of eggs laid was counted after single or dual knock-down of *E93* and *Kr-h1*, and dsGFP injected into the fifth instar nymph was used as a control. The number of eggs is also an important indicator of pest outbreaks. We counted and recorded the eggs laid by female brown planthoppers. The brown planthopper ovary during development was divided into five grades, as shown in Figure 2A. The results showed that the downregulation of *E93* significantly reduced the number of eggs laid (1.8 eggs/female, Figure 2B), and the downregulation of *Kr-h1* also significantly reduced the number of eggs laid (31.6 eggs/female, Figure 2B). However, the dual knock-down of *E93* and *Kr-h1* significantly increased the number of eggs laid compared to the knock-down of *E93* alone (Figure 2B). Moreover, we dissected and graded the ovaries at two days after adult emergence. All ovaries where only *E93* was downregulated were grade I (Figure 2C), and there were mainly grade II ovaries when only *Kr-h1* was downregulated (79.4%, Figure 2C). In both cases, the ovary developed slowly compared with the control (*p* < 0.001, Figure 2C). However, in the dual knock-down, ovary development increased significantly, 83.2% of grade I ovaries and 16.8% of grade II ovaries.

Taken together, dual knock-down increased ovary development and the number of eggs laid compared to the knock-down of *E93* alone, indicating that knock-down of *Kr-h1* partially recovered the deteriorating effect of the *E93* knock-down on ovary development.

### 2.3. E93 Interacts Directly with Kr-h1

**Figure 3:**
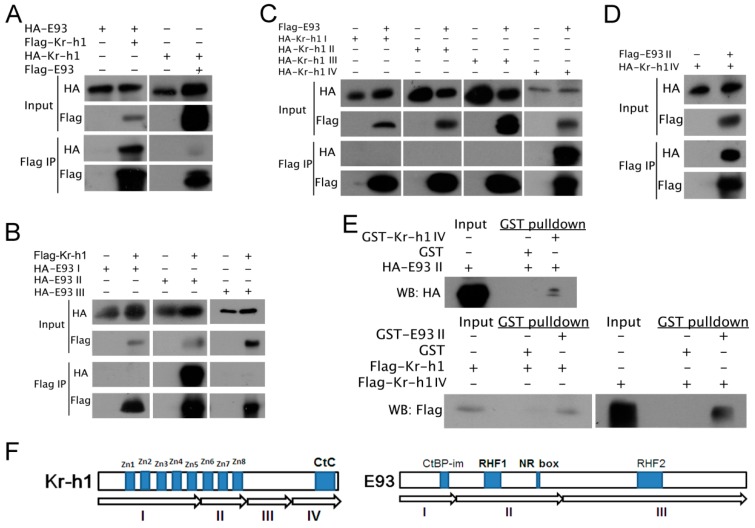
Interaction between E93 and Kr-h1. (**A**) E93 coimmunoprecipitated with Kr-h1. E93 and Kr-h1 were expressed separately and co-expressed. Immunoprecipitations from 293T cells were performed with anti-HA or anti-FLAG antibody, and HA/FLAG tag-fused E93 or Kr-h1 are shown as indicated. Beads with anti-FLAG antibody were used for Co-IP, and WB was used to check the Co-IP results. (**B**) Fragment II of E93 coimmunoprecipitated with Kr-h1. (**C**) WB results indicate that E93 interacts with fragment IV of Kr-h1. (**D**) WB results indicate that E93 II interacts with fragment IV of Kr-h1. The immunoprecipitation of (**B**–**D**) was performed as described in (**A**). (**E**) HA-E93 II, Flag-Kr-h1 and Flag-Kr-h1 IV were expressed in 293T cells, respectively, followed by GST fusion protein beads (GST-Kr-h1 IV-beads and GST-E93 II-beads) for pull down. The results of WB all showed that Kr-h1 IV and E93 II and E93 II and Kr-h1 interacted directly; (**F**) Schematic representation of Kr-h1 and E93 for identification of regions responsible for their protein–protein interaction. Fragment IV of Kr-h1 includes a recently identified C-terminal conserved (CtC domain) domain, fragment II of E93 contains domain RHF1 and NR box.

The effects of the dual knock-down of *E93*/*Kr-h1* on nymph development and ovary development indicated that *E93* and *Kr-h1* likely interact antagonistically. To understand the mechanism of antagonistic interaction between *E93* and *Kr-h1*, we cloned the full-length CDS of brown planthopper *E93* and *Kr-h1* into vectors for the co-immunoprecipitation of E93 and Kr-h1 tagged with either FLAG or HA separately. The results showed that E93 co-immunoprecipitated with Kr-h1, even after switching tags (Figure 3A), indicating that brown planthopper E93 interacts with Kr-h1 at the protein level (Figure 3A).

We analysed the predicted protein sequences of E93 and noticed that E93 has four important functional domains: CtBP-interacting motif (CtBP-im), nuclear receptor-box (NR-box), two helix-turn-helix DNA binding motifs of the pipsqueak family, RHF1 and RHF2. Additionally, the predicted Kr-h1 protein mainly contains eight zinc-finger domains in the N-terminus [6]. We divided E93 into three fragments, cloned these fragments into vectors for Co-IP and tested their interactions with Kr-h1. We also divided Kr-h1 into four fragments and cloned these fragments into vectors for Co-IP to determine which fragment is responsible for the interaction between E93 and Kr-h1 (Figure 3F). The results showed that fragment II of E93 co-immunoprecipitated with Kr-h1, indicating that fragment II of E93 is responsible for the interaction between E93 and Kr-h1 (Figure 3B). Furthermore, we tested the interaction between E93 and four fragments of Kr-h1. We found E93 co-immunoprecipitated with fragment IV of Kr-h1 (Figure 3C), which indicates that E93 II interacts with Kr-h1 IV (Figure 3D).

To further understand whether E93 II directly interacts with Kr-h1 IV, we expressed HA-E93 II in 293T cells. We expressed GST-fused fragment IV of Kr-h1 in *E. coli* and purified the fusion protein, followed by using GST fusion protein beads (GST-Kr-h1 IV-beads) for the pull-down assay. We also expressed Flag-Kr-h1 and Flag-Kr-h1 IV in 293T cells, followed by using GST fusion protein beads (GST-E93 II-beads) for the pull-down assay. Both experiments showed that the corresponding protein was pulled down (Figure 3E). Kr-h1 and E93 II, Kr-h1 IV and E93 II all showed strong and specific direct interactions (Figure 3E), further indicating that the potential interacting domain of E93 with Kr-h1 was located in the corresponding fragment. In summary, we inferred that the NR-box and RHF1 of E93 would be the candidate domains responsible for the protein–protein interaction between E93 and Kr-h1.

### 2.4. Sub-Cellular Localization of E93 and Kr-h1

**Figure 4:**
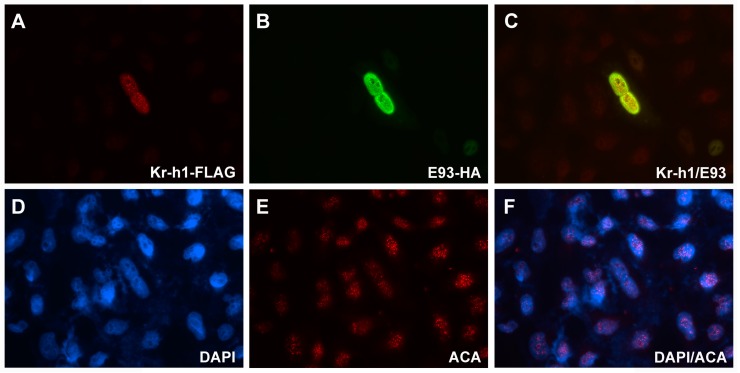
Co-localization of E93 and Kr-h1 in the nucleus (600× magnification). DAPI was used to stain the nucleus. Anti-HA and anti-FLAG were used to show the sub-cellular localization of E93 and Kr-h1, both of which were fused to tags separately. ACA was used to stain the centromere as an internal control. (**A**) FLAG-tagged Kr-h1; (**B**) HA-tagged E93; (**C**) Merge of FLAG-tagged Kr-h1 and HA-tagged E93; (**D**) DAPI; (**E**) ACA; (**F**) Merge of DAPI and ACA.

To determine the sub-cellular localization of E93 and Kr-h1, we co-transfected an HA-tagged E93 plasmid and a FLAG-tagged Kr-h1 plasmid into HeLa cells. Then, the primary antibodies anti-HA and anti-FLAG were added to bind with the fusion proteins. The results showed that these two proteins were co-localized in the nucleus (Figure 4B,C), indicating that they might function in transcriptional regulation, further suggesting that these two proteins may be related to transcriptional regulation.

### 2.5. Expression of E93 and Kr-h1 was Increased When Kr-h1 is Down-Regulated Compared to the Knock Down of E93 Alone

**Figure 5:**
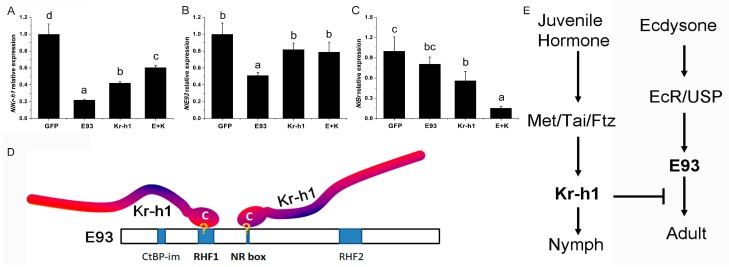
Expression of *E93*, *Kr-h1* and *Br-C* when *E93* and *Kr-h1* were downregulated. Transcriptional changes of *E93*, *Kr-h1* and *Br* in the single or dual knock down of *E93*/*Kr-h1* in the brown planthopper. Relative expression of *E93* (**A**), *Kr-h1* (**B**) and *Br-C* (**C**) is shown; (**D**) schematic model of the interaction between Kr-h1 and E93. Duncan’s multiple comparison was used, *p* < 0.05; (**E**) Scheme of JH signalling and ecdysone signalling, and the interaction of two pathways is mediated by the interaction between Kr-h1 and E93.

The most direct effect of the interaction between transcription factors is transcriptional regulation. To further evaluate the effect of the interaction between E93 and Kr-h1 on the transcriptional changes of *E93* and *Kr-h1*, we injected 5th instar nymphs with the dsRNA of *E93* and *Kr-h1* alone or in combination and measured the expression of *E93* and *Kr-h1* after three days in the single and dual knock-down brown planthopper. The expression of *E93* and *Kr-h1* was reduced significantly in the single knock-down of the corresponding gene (Figure 5A,B). However, we found that the expression of *E93* in the *Kr-h1* knock-down and dual knock-down brown planthopper was not changed significantly compared to that in the dsGFP control (Figure 5B), and the expression of *E93* was actually increased compared to that in the *E93* knock-down brown planthopper (Figure 5A,B), suggesting that the knock down of *Kr-h1* increased the expression of *E93* (Figure 5B). The expression of *Kr-h1* in *E93* knock-down cells decreased significantly (*p* < 0.05, Figure 5A). In the *Kr-h1* knock-down and dual knock-down, the expression of *Kr-h1* was also decreased compared to that in the control transfected with dsGFP but increased significantly compared to that in the *E93* knock-down (Figure 5A), which indicates the complex regulatory and possible feedback interaction on the expression of *Kr-h1*. In addition, the expression level of *Br* was decreased when *Kr-h1* was knocked down, and *Br* expression was even more significantly decreased when both *E93* and *Kr-h1* were knocked down (Figure 5C).

Taken together, these results suggest that the expression of *E93* is likely regulated directly by *Kr-h1*.

## 3. Discussion

The knock-down of *E93* in 3rd instar nymphs showed a 0% survival rate, which increased to 90% when knocked down at the 4th instar nymph stage and decreased to 50% when knocked down at the 5th instar nymph stage (Figure 1). The 5th nymph instar is the last nymphal stage and therefore is the most important stage for *E93*, which plays a crucial role in specifying metamorphosis, as previously shown in other insects [11,13,18,20]. For the high lethality in the 3rd instar nymph after *E93* knock-down, we surmise that this effect is a direct or indirect result of the multiple functions of *E93* required for the development of the nymph, such as apoptosis and autophagy [14,19]. Moreover, different nymph stages have different hormone titers. The juvenile hormone titer decreased during the 5th instar nymph stage, while the titer of ecdysone increased during this stage. We surmise that the 5th instar nymph is more sensitive to the change in *E93* expression level, resulting in a lower survival rate after RNAi than the 4th instar nymph. Therefore, in the 3rd instar nymph and the 5th instar nymph, when the expression level of *Kr-h1* that inhibits *E93* transcription was simultaneously down-regulated with *E93*, the survival rate was significantly higher than that of down-regulation of *E93* alone. In summary, our RNAi experiment suggested that *Kr-h1* and *E93* may interact antagonistically. Moreover, protein–protein interaction experiments showed that the brown planthopper E93 interacts directly with Kr-h1, while the expression of *E93* increased when *Kr-h1* was downregulated or both *Kr-h1* and *E93* were downregulated compared to the knock down of *E93* alone (Figure 5B), again indicating that these two proteins may interact antagonistically.

Both *E93* and *Kr-h1* are necessary for nymph development, metamorphosis, and adult reproduction. The role of *E93* and *Kr-h1* in ovary development is one of the key functions and is also a sensitive readout for evaluating the function and interaction of different genes or signalling pathways. Although the effect of the knock down of *E93* at the 5th instar nymph stage almost eliminated the reproductive ability of females, the dual knock down of *E93* and *Kr-h1* rescued part of the reproductive ability (Figure 2A). A similar “rescue” phenomenon was observed when we compared ovary development through ovary grading; dual knock-down increased the development speed of the ovary compared to that with the knock down of *E93* alone (Figure 2B). Moreover, we noticed that the “rescue” is only partial, which is possibly a result of the incomplete elimination of the activity of Kr-h1 by RNAi. In addition, the survival rate experiment showed that dsRNAs have different effects when used in different phases of development (Figure 1B), we surmise which might be caused by the variations of the hormone titers during the development.

Shinoda and colleagues used a reporter assay in a silkworm cell line and found that Kr-h1 binds to a Kr-h1 binding site (KBS) in the promoter region of *E93,* and the C-terminal conserved (CtC) domain of Kr-h1 is essential for the repression [23]. By amino acid sequence comparison, we found that the CtC domain is also conserved across hemi-metabolous insects, including the brown planthopper [6]. In this study, we found that the C-terminal of Kr-h1 (Kr-h1 IV) is responsible for the physical interaction between Kr-h1 and E93, indicating that the interaction between these proteins might be crucial for their biological function. Moreover, we found that the C-terminus of brown planthopper Kr-h1 (Kr-h1 IV) interacted with fragment II of brown planthopper E93 (E93 II). Interestingly, E93 II contains an RHF domain (RHF1) and a nuclear receptor interaction motif (NR-box). As mentioned above, the RHF domain is an HTH-DNA binding motif that interacts with DNA, and the NR-box is able to interact with nuclear receptors [17]. Therefore, we propose a more direct and specific model in which the C terminus of Kr-h1 inhibits the DNA binding motif of E93 and thereby inhibits its transcriptional activity (Figure 5D). This model is also complementary to the binding ability of Kr-h1 with the KBS region, which is located in the E93 promoter region, i.e., by binding with the KBS region to compete for the activation of E93 transcription, and additionally, binding with the E93 DNA-binding motif masked the DNA interacting interface of E93, which reduced its transcriptional activity and repressed adult metamorphosis (Figure 5D,E). Furthermore, the protein–protein interaction provided more specificity to the inhibitory activity of Kr-h1. Alternatively, by interacting with nuclear receptors, such as ultraspiracle (USP) or Ecdysone Receptor (EcR), Kr-h1 masked receptor activity and attenuated transcriptional activity (Figure 5D,E).

A previous study in *Tribolium castaneum* showed that the repression of *E93* transcription by Kr-h1 is crucial to the strong upregulation of Broad-complex (Br-C) [20], indicating that the interaction between E93 and Kr-h1 involves more key factors related to the JH and ecdysone signalling pathway. Moreover, the co-localization of E93 and Kr-h1 suggests possible functions and physical interactions between E93 and Kr-h1 and their transcriptional regulation of downstream genes. JH and ecdysone signalling are critical for the control of ovary development and pest populations. Our result is important for understanding the interaction between E93 and interacting proteins, which makes it possible to identify potential targets and develop new pesticides for pest management.

## 4. Materials and Methods

### 4.1. Insects

The insects maintained in our lab were originally a gift from Professor Zengrong Zhu (Zhejiang University, Zhejiang, China). The insects were raised at 28 °C, light:dark = 14:10 at 70–80% humidity using seedlings of the rice variety IIyou-023 (*Oryza sativa* L. cv.) cultured with nutrient solution [24].

### 4.2. Cloning of Genes for dsRNA Synthesis

Total RNA was extracted using the Trizol-based RNAiso Plus total RNA extraction kit (Takara, Dalian, China), and the Roche Transcriptor First-Strand cDNA Synthesis Kit (Roche Applied Science, Shanghai, China) was used to synthesize first-strand cDNA.

The brown planthopper homologue of *Kr-h1* was identified from previously published sequences of *N. lugens* [22]. The previously cloned *Kr-h1* full-length cDNA fragment was used as a template for dsRNA synthesis and amplified by PCR using Ex-Taq polymerase (Takara, Dalian, China). *E93* was identified from both the transcriptome sequence and the NCBI database (www.ncbi.nlm.nih.gov). The primers for cloning *E93* were designed and listed in Table 1. *E93* was cloned, confirmed by sequencing and used for dsRNA synthesis.

### 4.3. RNAi

dsRNAs of brown planthopper *Kr-h1* and *E93* were synthesized using the RiboMAX™ Large Scale RNA Production System-T7 (Promega, Madison, WI, USA). The synthesis procedure was the same as Technical Bulletin TB166 (Promega), except that different templates and primers were used for synthesizing different dsRNA (with 27 bases at 5′, Table 1).

Nymphs of the appropriate developmental stage were anaesthetized by CO_2_. A Nikon microscope and Narishige injection system (MN-151, Narishige Scientific Instrument Lab, Tokyo, Japan) were used for injection; 3rd, 4th and 5th instar nymphs were anaesthesized by CO_2_ and 0.1 μg (0.2 μL) dsRNA was injected into the abdomen of each nymph. The nymphs were recovered for 2 h after injection and cultured on rice seedlings. The same amount of dsRNA was used for the dual knock-down experiment (0.1 μg for each dsRNA) [25].

### 4.4. qRT-PCR

We collected the samples for analysis three days after dsRNA injection, and total RNA was extracted. First-strand cDNA was synthesized using the Roche Transcriptor First-Strand cDNA Synthesis Kit (Roche Applied Science, Shanghai, China).

cDNA was diluted 20 times after synthesis and used for qRT-PCR. Hieff^TM^ qPCR SYBR^®^ Green Master Mix (High Rox Plus) (Yeasen Biotech Co., Ltd., Shanghai, China) was used for qRT-PCR. We used a 25 μL reaction, and 2 μL of diluted cDNA was used as the template. Expression levels were compared using the 2^-∆∆*C*t^ relative expression method [26]. The reference gene for qRT-PCR was selected based on a previous study [27]. The primers used are listed in Table 1.

### 4.5. Egg Counting, Ovary Dissection, and Classification

The dsRNA-injected brown planthopper and the non-treated control (NC) were cultured using rice seedlings. After eclosion, the females were collected, and one female was paired with one male and cultured with rice seedlings. The rice seedlings were dissected for eggs every other day, and the number of eggs laid was then counted under the microscope and recorded. The dissections for ovary extraction were performed as follow: the brown planthopper females were anaesthetized by CO2 and were dissected in PBS with a pair of tweezers, one to hold the thorax and the other to hold the lower part of the abdomen to gently tear the abdomen apart and expose the ovaries. The ovaries were then mounted in glycerol for further observation and counting (the number of ovaries at different grades).

### 4.6. Construction of Plasmids

The procedure for the construction of the plasmid is amplification by PCR using the primers in Table 1, then digestion with the corresponding restriction enzymes, and cloning into the corresponding vectors. pXF6F (FLAG tag) and pXF4H (HA tag) were used for Co-IP, and pGEX4T-1 was used for GST pull-down.

For cloning full-length Kr-h1, primers 5′-CGGATCCTTCAATACGGAGGGAGGAGG-3′ and 5′-GGAA TTCTTAGGAGGCCTTGGCAT AGT-3′ were used. For cloning the N terminal fragment of Kr-h1 (fragment I), primers 5′-CGGGATCCTTCAATACGGAGGGAGGAGG-3′ and 5′-CGGGATCCTTAGTGACTCTT G ATGTGGC CTT-3′ were used. For cloning fragments II, III and IV, we used the following primers. II: 5′-GG AATTCTCT GAGCCGGCAGCACCGGC-3′ and 5′-CGGATCCTTGATGTTCGGATGCGG CTG-3′, III: 5′-GGAATTC AGAGCTGTGCCAATACCTAT-3′ and 5′-CGGATCCGCAGCTGGTA CTGATAGGCC-3′, IV: 5′-GGAAT TCATCCTCTGCCCGAGTGCCTG-3′ and 5′-CGGATCCTTA GGAGGCCTTGGCATAGT-3′. For cloning full-length E93, the primers 5′-GGAATTCGGGAGAACGAAATGGCGAGA-3′ and 5′-GCTCT AGACTATGACTCTTGCCGTTCTG-3′ were used. For cloning fragments I, II and III of E93, we used the following primers: I: 5′-GGAATTCGGGAGAACGAAAT GGCGAGA-3′ and 5′-GCTCTAGACTACAGA TCGAGAGGTGAGGGTG-3′, II: 5′-GGAATTCC CCGCTGGCTTCCAAGGTCT-3′ and 5′-GCTCTAGAC TATTGTTGTGGAATGTGATGCA-3′, III: 5′-GGAATTCCCTCCTCAACATACACTGGA-3′ and 5′-GCTC TAGACTATGACTCTTGC CGTTCTG-3′. The plasmids for GST pull-down were constructed using the primers 5′-GGAATTCATCCTCTGCCCGAGTGCCTG-3′ and 5’-CCGCTCGAGTTAGGAGGCCTTGGC ATAGT-3′ (IV of Kr-h1), 5′-GGAATTCCCCGCTGGCTTCCAAGGTCT-3′ and 5′-GCTCTAGACTAGTT CATTTTTCTTTCATCAA-3′ (II of E93).

### 4.7. Cell Culture

The 293T cells and HeLa cells were cultured in DMEM containing 10% foetal calf serum at 37 °C, 5% CO_2_, and constant humidity. Penicillin and streptomycin were added at a final concentration of 1% to prevent cell contamination.

### 4.8. Transiently Transfects Plasmid into Cells

Cells cultured in a 6-well plate with medium containing no antibiotics were used for transfection. Transfection was performed when the cell density reached approximately 50–80%. FuGENE 6 transfection reagent (Promega, Madison, WI, USA) was used, and the transfection was carried out as described in the protocol by Promega (TM350).

### 4.9. GST Pull-Down

The plasmid of GST fusion protein was transformed into strain BL21 and cultured until OD 600 = 0.6–0.8. Then, IPTG was added to a final concentration of 0.1–0.4 mM and induction was performed overnight at 16 °C. The bacteria were collected and re-suspended as a 25 mL lysate. The cells were sonicated and centrifuged, and the supernatant was incubated with Glutathione Sepharose 4B beads (GE Healthcare, Barington, IL, USA) for 4 h at 4 °C. Then, the supernatant was discarded, and the 4B beads were washed 3–5 times with lysate buffer. The beads combined with the GST fusion protein were used for the pull-down experiment.

The 293T cells were lysed and centrifuged. The supernatant was pre-incubated with empty sepharose beads for 30 min. Then, 60 µL of protein supernatant was taken as input after centrifugation. The remaining supernatants were equally divided, incubated with GST beads conjugated with 2–3 µg of fusion protein or beads with only GST protein for 5 h. After centrifugation for 1 min, the supernatant was discarded, and the lysate was washed. The remaining steps were the same as immunoprecipitation (IP).

### 4.10. Co-Immunoprecipitation (Co-IP)

The 293T cells cultured in a 10 cm dish were used for transfection. The cell suspension was collected, lysed and centrifuged. The supernatant was used for Co-IP. GammaBind Protein G Sepharose beads (GE healthcare, 10 µL), which had been previously prepared with cell lysate and pre-incubated for 30 min, was added to the supernatant. Twenty microliters of the protein supernatant was taken as input after centrifugation. The rest of the supernatant was placed into an EP tube, and 2–3 µg of antibody (anti-FLAG, Sigma, Shanghai, China; anti-HA, Covance, NJ, USA) was added. The supernatant was incubated at 4 °C for 3 h and then added to GammaBind Protein G Sepharose beads (GE Healthcare, the USA, 20 µL) for 1 h, or 5 µL of antibody-bound beads was added and incubated for 4 h at 4 °C. After centrifugation, the supernatant was aspirated, and 1 mL of lysate was added, and the beads were washed three times. Then, 50 µL of 2× SDS protein loading buffer was added, and the samples were heated at 95 °C for 10 min, followed by immunoblotting.

### 4.11. Western Blotting

The prepared protein sample and protein marker were separated by SDS-PAGE. The protein was transferred to a PVDF membrane pretreated with methanol at a constant flow of 350–380 mA for 2 h on ice. The PVDF membrane was washed 3 times with PBST buffer, and the primary antibody (anti-FLAG, Sigma, Shanghai, China; anti-HA, Covance, USA) was added and incubated overnight at 4 °C. The PVDF membrane was washed 3 times with PBST buffer for 10 min each time. An HRP-conjugated secondary antibody (anti-mouse/rabbit IgG HRP-linked antibody, CST, Danvers, MA, USA) prepared in PBST buffer was added and the blot was incubated for 1 h. The PVDF membrane was washed 3 times with PBST buffer for 10 min. An HRP substrate was added for development.

### 4.12. Cellular Localization Experiment

The FLAG- and HA-tagged plasmids were co-transfected into HeLa cells. The cells were fixed with 4% formaldehyde for 30 min and washed with PBST for 3 times (10 min/wash). The primary antibody (anti-FLAG, Sigma, USA; anti-HA, Covance, USA) and secondary antibody (Donkey anti-mouse IgG-Alexa Fluor 488, Donkey anti-rabbit IgG-Alexa Fluor Cy3, Jackson ImmunoResearch, West Grove, PA, USA) were used. Each antibody was incubated for 1 h and washed with PBST for 3 times (10 min/wash). DAPI was used for staining of the nucleus and ACA (HCT-0100, Immunovision, Springdale, AR, USA) was used as a control.

### 4.13. Imaging and Statistical Analyses

Images of the ovaries and cellular localization were taken using a Nikon fluorescence microscope (Eclipse 80i, 4X) with NIS-Elements. The images were processed with Adobe Photoshop CS5 software. The statistical analyses were carried out using SPSS 20.0 and Origin 10.0. The statistical methods used are described in the figure legends.

## 5. Conclusions

Brown planthopper *E93* and *Kr-h1* have antagonistic effects on regulating metamorphosis and ovary development. Kr-h1 and E93 undergo strong and specific interactions, and the nuclear receptor interaction motif (NR-box) and helix-turn-helix DNA binding motifs of the pipsqueak family (RHF1) are candidate domains responsible for the protein–protein interaction between E93 and Kr-h1. 

## Figures and Tables

**Table 1:** Primers for cloning, QRT-PCR and dsRNA synthesis.

Name	Sequence (5′-3′)
E93F	ATGGACAGCAAGGCCTGGCATC
E93R	CTATGACTCTTGCCGTTCTGATC
E93QF	AACAACCTCCCGAAATGCAT
E93QR	TGCATATGATGGTGGTGGTG
KrhQF	TGATGAGGCACACGATGACT
KrhQR	ATGGAAGGCCACATCAAGAG
RP15	CCGATCGTGTGGCGTTGAAGGG
RP15	ATGGCCGACATTCTTCCAGGTCC
dsGFP	TAATACGACTCACTATAGGGAGACCACGGGCGAGGAGCTGTTCACCG
dsGFP	TAATACGACTCACTATAGGGAGACCACGCAGGACCATGTGATCGCGC
dsKrF	TAATACGACTCACTATAGGGAGACCACGTGGGGTTCAGTCCTGAGGA
dsKrR	TAATACGACTCACTATAGGGAGACCACCAGTCGAACACACACCGGAC
dsE93F	TAATACGACTCACTATAGGGAGACCACGCCAGCTTACATGACGAAGA
dsE93R	TAATACGACTCACTATAGGGAGACCACCAGAGTGCAGGATGGATGAC
